# The role of microRNAs in oral lichenoid disorders. Systematic review

**DOI:** 10.4317/medoral.21819

**Published:** 2017-08-16

**Authors:** Amaia Setién-Olarra, María-Luisa Gainza-Cirauqui, José-Manuel Aguirre-Urizar, Xabier Marichalar-Mendia

**Affiliations:** 1 Oral Medicine and Pathology, Department of Stomatology II, UFI 11/25, University of the Basque Country (UPV/EHU), Leioa, Spain; 2 Department of Dental Surgery, Faculty of Dental Surgery, University of Malta, Msida, Malta

## Abstract

**Background:**

Certain changes in the microRNA expression are considered to be associated with chronic inflammatory processes and with the malignant transformation of oral potentially malignant disorders. The purpose of this systematic review is to update the existing data on the aberrant microRNA expression profiles identified in oral lichenoid disease (OLD).

**Material and Methods:**

A search in PubMed-Medline and Scopus was performed on the English literature published between 2010 and August 2016 using the following keywords: oral lichenoid disease, oral lichen planus and microRNA.

**Results:**

Originally, 25 articles were considered, of which 12 case-control articles were selected according to the inclusion/exclusion criteria.

**Conclusions:**

OLD seems to have altered microRNA expression profile. Certain altered microRNAs (146a, 155) may be useful as biomarkers for this disorder. More studies including larger number of cases are needed in order to study further on the biological processes and on the regulation pathways of these altered microRNAs.

** Key words:**microRNAs, oral lichenoid disease, oral lichen planus.

## Introduction

Oral lichenoid disease (OLD) has been considered typically as a chronic inflammatory disorder with an immunological base that can be present in 2% of the population and mainly affecting women (1-3). This disease is considered an oral potentially malignant disorder with a low percentage of malignant transformation, although varying widely between 0.4 and 6.5% (2). To date, the data is unreliable in predicting the risk of malignant transformation of this disorder and in establishing effective measures to avoid it (4,5).

MicroRNAs are non-coding RNA molecules that regulate the gene expression post-transcriptionally through imperfect binding to the 3` region of the messenger RNA in a partially complementary manner causing the transcriptional repression or the direct degradation of the mRNA (6,7). It is estimated that the nearly 2000 microRNAs described can regulate the expression of 60% of the human genes and regulate at the same time biological processes such as growth, differentiation and cell death (8), so its deregulation is closely involved in several biological and molecular processes that drive tumorogenesis, acting as oncogenes or as tumor suppressor genes (9). Changes in the microRNA expression profile during the process of malignant transformation of premalignant lesions to oral cancer have been described (10-12).

Proper regulation of microRNA expression is important in maintaining normal immune fuctions and preventing autoimmunity (13). Moreover, are reported to be valuable biomarkers for human inflammatory and autoimmune diseases such as oral lichenoid disease (OLD) (13). According to Setién-Olarra *et al.* (14) it seems that the microRNA expression profile associated with OLD reflects a clear immune component of the disease, together with the key role played by cell proliferation processes. More precisely, a relation between the T lymphocyte-mediated immune response and the changes in the expression of certain microRNAs has been recognized (11,15). Nonetheless, there is limited information on the microRNA expression profiles in OLD.

The aim of this review is to update the existing data on the aberrant microRNA expression profile in oral lichenoid disease.

## Material and Methods

- Search strategy

The study consisted on a literature search on the microRNA expression changes in oral lichenoid disease (OLD) performed in PubMed-Medline and Scopus databases in articles published between 2010 and August 2016. The search included articles published in the English language using the following keywords: oral lichenoid disease, oral lichen planus, oral precancer and microRNA. These terms were linked in different combinations by using the Boolean operators “AND” and “OR”. After eliminating the duplicates, the articles were filtered according to the following inclusion and exclusion criteria.

- Inclusion criteria: Case-control studies in English, with well defined diagnostic criteria both on a genomewide approach and on a candidate gene approach, analyzed in (a) fresh tissue samples, (b) formalin-fixed paraffin-embedded (FFPE) samples, and (c) biological fluids.

- Exclusion criteria: Articles published in any language other than English, systematic reviews, articles not available in the data-bases, articles presenting bioinformatics results exclusively.

## Results

**Figure 1:**
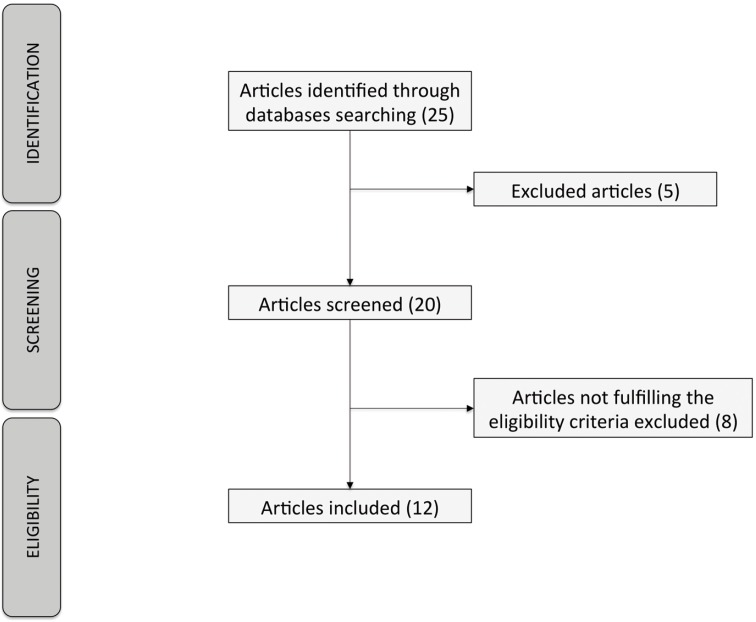
Flowchart describing the search strategy.

From an initial search, we obtained 25 articles. Subsequently, and after analysing them with our inclusion and exclusion criteria, 20 articles were selected for our study. After reading the abstracts, 8 articles were excluded for not fulfilling the inclusion criteria. Finally, 12 articles were selected for the systematic review (Fig. 1).

The microRNAs identified as altered for this disorder from these 12 selected articles, their levels of expression, the type of sample analyzed, their targets and the article where the alteration is described are shown in  Table 1.

**Table 1:**
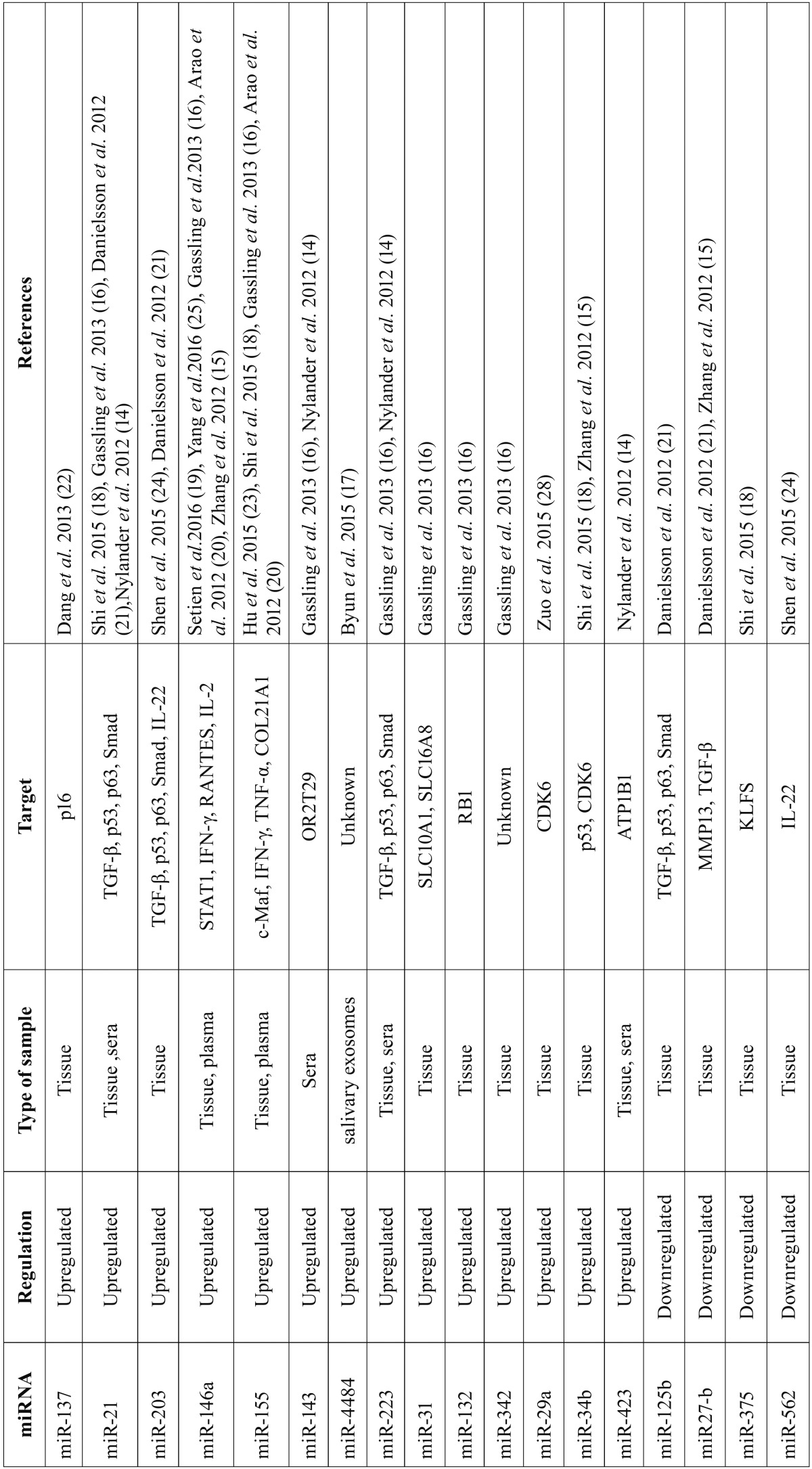
Aberrant expression of specific microRNAs in lichenoid disorders.

- Description of the studies

All of the articles selected for this systematic review are case-control studies and are either genomewide approach or candidate gene approach studies. Of the 12 articles, 6 (50%) were genomewide approach studies (13,14,16-19). In 5 of them (14,16-19), the expression profile of 768 microRNAs was analyzed with arrays, while the sixth article analyzed the profile of 667 microRNAs (13). From all the articles, 168 microRNAs were identified as aberrant in OLD. Furthermore, 2 of the studies (14,18) performed the validation of 17 microRNAs on an independent set of samples.

On the other hand, the remaining 6 articles (20-25), that had a candidate gene approach, analyzed the expression profile of 9 specific microRNAs.

## Discussion

The discussion has been divided according to the different types of samples studied.

- A. microRNA expression profile in oral tissue samples

In 2016, Setién-Olarra *et al.* (14) analyzed, in the highest number of OLD samples to date, the expression profile of 768 mature (16 OLD, 8 OSCC and 8 Controls). Of these, 20 microRNAs were deregulated, of which, the 13 best positioned (microRNA-150, microRNA-142, microRNA-146a, microRNA-223, microRNA-7, microRNA-339, microRNA-342, microRNA-146b, microRNA-140, microRNA-1247, microRNA-152, microRNA-625, microRNA-629) were successfully validated in an independent set formed by 91 samples (36 OLD, 28 OSCC and 27 Controls). According to Setién-Olarra *et al.* (14) the aberrant expression profile associated with OLD would reflect a clear immunological component of the disease; furthermore, the cellular proliferation processes and the response to the organic substances would play an important role in this pathology. In this regard, in 2013, Gassling *et al.* (17) studied the microRNA expression profile in 7 patients with oral lichen planus (OLP) and 7 control patients, identifying 24 deregulated microRNAs and 2694 transcribed mRNAs of which, most are functionally associated with inflammatory or premalignant events. These results suggest that the microRNAs regulate pathways involved in this disease and, therefore, may become an important therapeutic tool (17).

Furthermore, Zhang *et al.* (13) analyzed the global microRNA expression pattern in oral biopsies on three patients diagnosed with oral lichen planus and 3 control patients, identifying 46 differentially expressed microRNAs from the 667 analyzed. In addition, the aberrant expression of the microRNA-27b was validated in 2 independent sets of samples. In detail, Zhang *et al.* (13) noticed low levels of microRNA-27b in OLP biopsies, and suggested that the underexpression of this microRNA may be a predisposing factor of presenting this disease. It is known that this microRNA is able to intervene in the regulation of cell differentiation, in the immune response and in chronic inflammation (26,27).

To date, microRNA-146a has been described in genomewide approach studies as the only microRNA with a clear overexpression in this disease (13,14,17). These results are in agreement with previous candidate-microRNA studies (20,25,28).

In 2012, Arão *et al.* (20) confirmed that the microRNAs, microRNA-146a and microRNA-155, that affect the regulation of the immune response, including the kinase- and transcription factor-mediated signalling pathways, were overexpressed in OLP. Subsequently, in the year 2016, Yang *et al.* (25) analyzed the levels of microRNA-146a, in tissue and blood samples of 16 patients diagnosed with OLP and 9 controls in order to study the expression of this microRNA in CD4+ cells in peripheral blood. These authors (25) were incapable of finding differences in the expression of miR-146a in the two types of sample of the CD4+ cells in peripheral blood, but observed differences in tissue samples, where they could confirm the overexpression of miR-146a in OLP. Additionally, they identified a greater overexpression of this microRNA in erosive OLP samples when compared to non-erosive OLP samples, therefore concluding that miR-146a may have a role in the malignant potential of erosive OLP, although a further in-depth study would be required. In this sense, studies show that microRNA-146a is overexpressed in some inflammatory diseases such as psoriasis (29), rheumatoid arthritis (30), osteoarthritis (31), as well as in oral lichenoid disease (13,14,17,20,25). All this suggests that microRNA-146a may play an important role in the physiopathology of these immunologically-based inflammatory conditions (32). Nonetheless, the true function and its mechanisms of action are yet to be clarified.

On the other hand, Hu *et al.* (23) described the overexpression of miR-155 by analyzing the relation between this microRNA and the cytokines from peripheral blood samples of 17 patients diagnosed with erosive OLP, 10 patients diagnosed with non-erosive OLP and 13 controls. These authors (23) observed a relation between the overexpression of this microRNA in peripheral blood of erosive oral lichen planus and the severity of the lesion. Furthermore, they detected a positive miR-155- interferon-γ feedback loop in erosive oral lichen planus CD4+ cell samples which, according to these authors (23), may contribute to the immune re-sponse dominated by the Th1 type cells in this type of lesions.

Shi *et al.* (19) analyzed the paired microRNA-mRNA expression profile with next-generation sequencing in samples of normal adjacent tissue, OLP and OSCC of 2 patients and obtained 31 overexpressed and 7 underexpressed microRNAs. Among these 38 microRNAs, microRNA-375 presented the most significant aberrant expression. They observed that the expression of this mi-croRNA was decreasing as the normal tissue transformed and malignant features appeared, thus these authors (19) suggest that, in the process of a malignant transformation, microRNA-375 would be capable of regulating cell proliferation aided by KLFS, one of its potential targets. The strength of the Shi *et al.* (19) study relies on the analysis of paired premalignant and cancer samples from the same patients, providing integrated profiles of mRNAs, thus, new options for early diagnosis of oral cancer.

On the other hand, in 2012, Danielsson *et al.* (21) selected 3 microRNAs (miR-21, miR-125b, and miR-203) with a connection with p53, DNp63, and TGF to perform a candidate gene study. These authors (21) analyzed the levels of expression of these 3 microRNAs in oral biopsies of 20 patients diagnosed with OLP and 20 healthy controls, observing an overexpression both of microRNA-21 and of microRNA-203 and an underexpression of microRNA-125b. Furthermore, they found that the p53 and DNp63 targets were underexpressed in lichen planus samples when compared to the healthy controls. The overexpression of mi-croRNA-21 has also been described in other studies on the aberrant microRNA expression profile in OLP (16,17) and even in oral leukoplakia, and in inflammatory diseases of the skin such as psoriasis and atopic eczema (10,30). The evident overexpression of microRNA-203 in OLP was also described by Shen *et al.* (24) These authors (24) studied 50 oral biopsies of patients diagnosed with OLP and 19 healthy patients showing that the overexpression of microRNA-203 and the underexpression of microRNA-562 and its probable target, interleukin-22, molecule involved in chronic inflammation and autoimmunity (33), play an important role in the pathogenesis of OLP. In this sense, these results (24) reinforce the hypothesis that OLP is an oral mucosal disorder of an eminently inflammatory chronic nature and immunological basis.

Finally, in 2013, Dang *et al.* (22) studied the epigenetic changes in the promoter of microRNA-137 in 20 OLP tissue samples, 12 OSCC and 10 controls and observed methylation in both microRNA-137 and p16 protein in OLP patients although at a lesser extent than in OSCC patients. Furthermore, they observed an association of this methylation with the epithelium in the OLP cases, which may indicate that the origin of the initial malignant process is in the epithelium and not in the connective tissue.

- B. microRNA expression profile in body fluids samples

Specifically, biopsies are recommended in order to arrive to a definitive diagnosis of OLP, as well as for subsequent histological and molecular analysis (33). Still, there are studies that consider of interest the use of biological fluids such as saliva and blood in the detection of alterations in the microRNA expression profiles as it would enable to obtain useful information through a simple and minimally invasive technique (34,35). Furthermore, non-invasive methods are more acceptable for patients diagnosed with OLP and requiring follow-up (18). In this regard, several studies have been published on the microRNA expression profile in OLP using biological fluid samples (16,18,23,25).

In particular, Byun *et al.* (18) analyzed the profile of microRNAs of salivary exosomes of 16 patients diagnosed with OLP and 8 control patients. They selected exosomes as the analysis sample since these are lipoprotein vesicles secreted by cells and protected from degradation (35,36), that prevent from false positives as they are free from polluting elements from saliva such as microRNAs derived from dead cells or other nucleic acids derived from pro-inflammatory cells (35). These authors (18) detected high levels of microRNA-4484 in samples from OLP patients. This microRNA has been previously associated with the immune response against pathological stimuli (37) and is thought to be a good identification biomarker for this pathology, although a greater number of studies are required in order to spot its potential targets (18).

On the other hand, through serum, Nylander *et al.* (16) analyzed the aberrant microRNA profile in 30 patients diagnosed with multifocal lichen planus and 10 control patients. For these authors (16), the possibility of using serum to perform these microRNA analyses is an advantage not only for its non-invasive nature but also for its “unlimited” nature. In the study, they detected 15 differentially expressed microRNAs, of which, the 3 overexpressed microRNAs that showed greater differences in their expression (miR-21, miR-223 and miR-143) had a connection with OSCC. Consequently, these authors (16) suggest that these 3 microRNAs may play a key role in the possible malignant transformation of OLP.

## Conclusions

The real etiology of oral lichenoid disease (OLD) and its potential to present a premalignant phenotype are currently unknown (2,3). Nonetheless, it has been suggested that microRNAs may be used in predicting the malignant transformation of oral potentially malignant disorders such as OLD (14,16,19). Although there is yet much to figure out from the role of microRNAs in this disorder, the altered microRNA expression profile seems to be different from controls. Furthermore, certain microRNAs, such as miR-146a and miR-155, may be useful as biomarkers for this disorder. However, more studies that confirm these results by analyzing the microRNA-mRNA profile in fresh tissue samples are required in order to identify both the microRNA-mRNA regulatory modules associated with deregulated microRNAs as well as the biological processes associated to these modules through an in silico functional analysis (14).
